# Cobalt Oxide-Containing Glaze/CaAlg Hydrogel Membrane for Degradation of Orange G via Peroxydisulfate Activation

**DOI:** 10.3390/gels12070645

**Published:** 2026-07-19

**Authors:** Bin Zhang, Minglin Wang, Yawen Liu, Jiabao Cui, Kongyin Zhao

**Affiliations:** 1 State Key Laboratory of Advanced Separation Membrane Materials, Tiangong University, Tianjin 300387, China; zhangbin@tiangong.edu.cn (B.Z.); 2331020438@tiangong.edu.cn (M.W.); 2531020456@tiangong.edu.cn (Y.L.); 2530020253@tiangong.edu.cn (J.C.); 2 Interdisciplinary Research Center for Advanced Textile Composites, College of Arts, School of Textile Science and Engineering, Tiangong University, Tianjin 300387, China

**Keywords:** glaze, CaAlg hydrogel membrane, advanced oxidation, degradation, orange G

## Abstract

The sustained expansion of printing and dyeing operations has led to the discharge of large volumes of organic wastewater containing dyes. The resulting environmental pollution demands urgent solutions, making the development of efficient and eco-friendly methods for the elimination of dyeing wastewater critically important. The combination of alginate hydrogel membranes with advanced oxidation processes (AOPs) for water purification represents an emerging approach in the current field of water treatment. In this study, a calcium alginate membrane was loaded with a glaze containing highly active cobalt oxide to fabricate a glaze-calcium alginate (Glaze-CaAlg) composite membrane. The membrane achieved stable degradation of Orange G dye under optimal conditions, and a series of tests were conducted under varying conditions using pollutant concentrations close to those found in real water bodies. Under optimal conditions (glaze loading = 2.5 mL, PMS = 0.3 mmol/L, cross-flow filtration mode), the membrane achieved a 93.7% degradation efficiency of Orange G (10 ppm) within 45 min, with hydroxyl radicals (·OH, ~79%) identified as the predominant reactive species. The Glaze-CaAlg membrane also exhibited excellent reusability, maintaining a degradation efficiency of over 80% for Orange G across five consecutive cycles. Furthermore, sodium citrate was employed to react with the Glaze-CaAlg membrane, enabling the recovery and secondary application of the glaze. Membranes re-fabricated from the recovered glaze showed mechanical strength and catalytic efficiency comparable to those of the pristine membrane. The Glaze-CaAlg membrane possesses high catalytic activity and good stability. This work offers a sustainable, cost-effective, and recyclable catalytic membrane that converts a traditional ceramic material into an advanced functional material for wastewater remediation, with great potential for practical application in the treatment of refractory organic pollutants.

## 1. Introduction

The rapid expansion of the printing and dyeing industry has exacerbated water environment pollution [1,2]. The extensive use of organic additives such as azo dyes, dispersants, and fixing agents poses a serious challenge to aquatic ecosystems [3]. Although azo dyes are indispensable in textile printing and dyeing, their stable molecular structure makes them resistant to natural degradation once released into water bodies [4]. Even trace amounts of residual dyes can accumulate through the food chain and induce teratogenic and carcinogenic risks [5]. Orange G (OG), a typical azo dye, provides efficient coloration and improves color fastness; however, the intermediates generated from its reductive cleavage are highly toxic, and OG has been included in the priority control pollutant lists of many countries [6,7]. Currently, the removal of azo dyes from aquatic environments mainly relies on adsorption and biodegradation methods [8]. Common engineering strategies include activated carbon adsorption and alternating aerobic–anaerobic biological treatment [9]. Nevertheless, adsorption only transfers pollutants from one phase to another, and the spent adsorbent may cause secondary pollution [10]. Biodegradation is limited by the bio-recalcitrant nature of dye molecules, leading to prolonged hydraulic retention times, and some metabolites still retain genotoxicity [11]. Therefore, developing efficient and thoroughly mineralizing treatment technologies for printing and dyeing wastewater is of great research significance.

Organic dyes and other organic contaminants present in printing and dyeing wastewater can be effectively removed by advanced oxidation processes (AOPs), particularly persulfate-based degradation technologies [12]. The core mechanism involves the activation of persulfates (e.g., peroxymonosulfate, PMS) via external energy or chemical excitation to generate reactive oxygen species (ROS), primarily sulfate radicals (SO_4_^−^·) and hydroxyl radicals (·OH) [13]. These radicals, possessing high oxidation potentials, efficiently attack chromophoric groups and aromatic ring structures in dye molecules, leading to bond cleavage, ring opening, and eventual mineralization into CO_2_ and H_2_O, or conversion into low-toxicity, readily biodegradable small-molecule intermediates [14,15]. Among various activation strategies, transition metal activation (e.g., using Co^2+^, Fe^2+^, Cu^2+^) has attracted extensive attention due to its mild reaction conditions and high radical yield [16]. These metal ions can catalyze the decomposition of PMS, enabling sustained radical generation [17]. Notably, in heterogeneous activation systems, supported transition metals (or their oxides) release active ions at a controlled rate while regenerating active sites through surface electron transfer, thereby significantly extending the lifespan of the oxidant and achieving deep removal of total organic carbon (TOC) from printing and dyeing wastewater [18,19]. Therefore, promoting the large-scale application of PS-AOPs technology in actual printing and dyeing wastewater treatment represents an effective approach to developing novel water treatment technologies that are highly efficient, stable, cost-effective, and environmentally friendly.

Blue-and-white glaze, the primary glaze used in the production of blue-and-white porcelain, is a quintessential representative of Chinese ceramic art [20]. In this glaze, cobalt oxide serves as the main colorant, and it is rich in oxides of Co, Mn, and Fe. In addition to the aforementioned oxides, the blue-and-white glaze also contains potash feldspar, quartz, and kaolin. Among these, cobalt is the major active center for activating peroxymonosulfate (PMS) in our study. Co^2+^ exhibits particularly outstanding activation efficiency and is recognized as the homogeneous catalytic center with the greatest potential for practical applications [21]. This process achieves near 100% atom utilization, markedly enhancing the catalytic degradation rate per unit mass of metal [22,23].

In this study, loading a cobalt oxide-containing glaze onto the hydrogel interface represents a practical application of PS-AOPs. The abundant cobalt oxide particles serve as the core component for fabricating the catalytic membrane, while the multiple metal species (Co, Mn, Fe) in the glaze form a multimetallic synergistic catalytic network [24]. The alginate, derived from natural brown algae, combines environmental friendliness with cost-effectiveness [25]. Employing the hydrogel membrane as a support for the glaze overcomes the preparation and application challenges of traditional powdered glaze catalysts, such as agglomeration, non-reusability, difficulty in recovery, and poor dispersibility. During the experiment, the pollutant-containing solution continuously permeated through the hydrogel membrane under a cross-flow filtration pressure field, achieving degradation efficiencies far superior to those of powdered glaze catalysis.

In the glaze-calcium alginate composite membrane (Glaze-CaAlg membrane), Co^2+^ exhibits the highest intrinsic activation efficiency toward peroxymonosulfate, enabling rapid generation of sulfate radicals. The variable valence properties of Mn and Mn (II)/Mn (IV) and Fe (II)/Fe (III) facilitate electron transfer, assist in the continuous regeneration of Co active sites, and simultaneously inhibit the deactivation of single-metal valence states [26,27]. Furthermore, the inert framework components of the glaze—such as potash feldspar, quartz, and kaolin—not only improve the thermal stability and mechanical strength of the composite membrane but also maintain the structural integrity of the membrane during hydrogel swelling, alleviating the detachment of active components commonly observed in conventional pure hydrogel membranes due to swelling. This greatly reduces the leaching risk of cobalt, manganese, and iron ions into the aqueous phase, allowing the composite membrane to sustain long-term catalytic activity in continuous flow reactions. The abundant carboxyl and hydroxyl groups of alginate further anchor the glaze particles through coordination, achieving uniform dispersion of the catalyst within the three-dimensional hydrogel network and preventing agglomeration. This composite system integrates the high organic pollutant degradation efficiency of advanced oxidation, the sustained activity of multimetallic synergistic catalysis, and the structural stability and low metal leaching characteristics provided by the glaze support, offering a recyclable, anti-deactivation, and environmentally friendly catalytic membrane material for advanced treatment of printing and dyeing wastewater.

## 2. Results and Discussion

### 2.1. SEM and EDS Analysis of Glaze and Glaze-CaAlg Membrane

**Figure 1:**
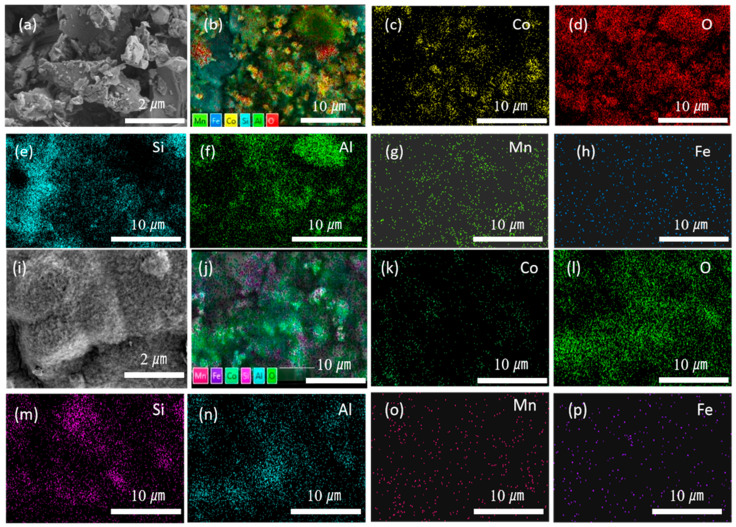
SEM image of the Glaze (**a**), EDS spectra of Glaze (**b**–**h**), SEM image of the Glaze-CaAlg membrane (**i**), and EDS spectra of Glaze-CaAlg membrane (**j**–**p**).

Figure 1 presents the microscopic morphology and elemental distribution of the glaze particles and the Glaze-CaAlg membrane. Specifically, Figure 1a–h show the SEM images and corresponding EDS elemental maps of the raw glaze material. Figure 1a reveals that the glaze particles exhibit irregular blocky shapes with relatively dense surfaces, and their particle sizes range from several microns to over ten microns.

Figure 1b–h demonstrate that characteristic elements such as Co, Si, Mn, and Fe are uniformly distributed within the particles. The presence of silicon (Si) signals is consistent with the high content of quartz and potash feldspar in the glaze. Cobalt, manganese, and iron originate from cobalt oxide, manganese oxide, and iron oxide components, respectively, and the overlapping distribution regions of these three elements indicate that the transition metal oxides are uniformly blended within the glaze. Figure 1i–p present the SEM images and EDS elemental mapping of the Glaze-CaAlg membrane. The membrane surface exhibits a continuous hydrogel matrix, within which the glaze particles are completely encapsulated by calcium alginate and uniformly dispersed throughout the three-dimensional network. Compared with the pure glaze, the elemental distribution in the membrane is more uniform.

**Figure 2:**
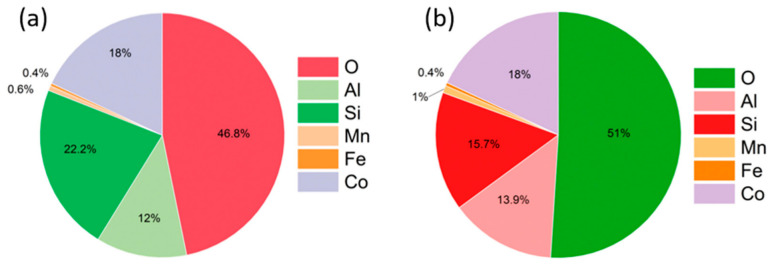
Element content in glaze particles (**a**) and element content in Glaze-CaAlg membrane (**b**).

Figure 2 presents the quantitative EDS analysis results of the major elements in the glaze and the composite membrane. Figure 2a shows that the contents of O, Si, and Co in the glaze particles are 46.8%, 22.2%, and 18.0%, respectively, along with minor amounts of Al (1.0%), Mn (0.6%), and Fe (0.4%). Figure 2b indicates that in the Glaze-CaAlg membrane, the O content increases to 51.0%, the Si content is 15.7%, while the relative contents of Al and Co are 13.9% and 18.0%, respectively. Combining the SEM images, EDS mapping, and quantitative results, it is confirmed that the glaze particles have been successfully and uniformly loaded into the calcium alginate hydrogel membrane, and the active metal components remain stable after incorporation.

### 2.2. XPS Analysis of Glaze and Glaze-CaAlg Membrane

To identify the surface oxidation states of the metal species in the Glaze and Glaze-CaAlg membrane, XPS analysis was performed.

**Figure 3:**
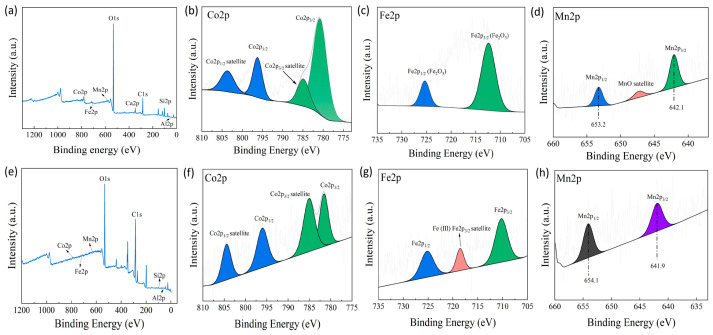
XPS full spectrum (**a**), Co2p spectrum (**b**), Fe2p spectrum (**c**), and Mn2p spectrum (**d**) of glaze. XPS full spectrum (**e**), Co2p spectrum (**f**), Fe2p spectrum (**g**), and Mn2p spectrum (**h**) of Glaze-CaAlg membrane.

As shown in Figure 3b, the Co 2p spectrum exhibits two major peaks at 780.8 eV (Co 2p_3_/_2_) and 803.7 eV (Co 2p_1_/_2_), indicating the existence of Co^2+^. The Co species are primarily present in the divalent state, which is favorable for PMS activation. In contrast, the Fe 2p spectrum displays peaks at 725.2 eV and 712.4 eV, corresponding to Fe(III). As shown in Figure 3f–g, the valence states of Co and Fe in the Glaze-CaAlg membrane are consistent with those of the glaze. Figure 3d indicates that the Mn element in the glaze is in a divalent state. The characteristic peaks are located at 653.2eV and 642.1eV, and MnO satellite peaks appear. Figure 3h shows that Mn element in the film displays peaks at 654.1 eV and 641.9 eV, respectively. After membrane formation, Mn remains in a divalent state.

Mixing glaze into calcium alginate film does not alter the chemical valence state of its main metal elements.

### 2.3. Analysis of Mechanical and Catalytic Properties of Different Glaze Contents

**Figure 4:**
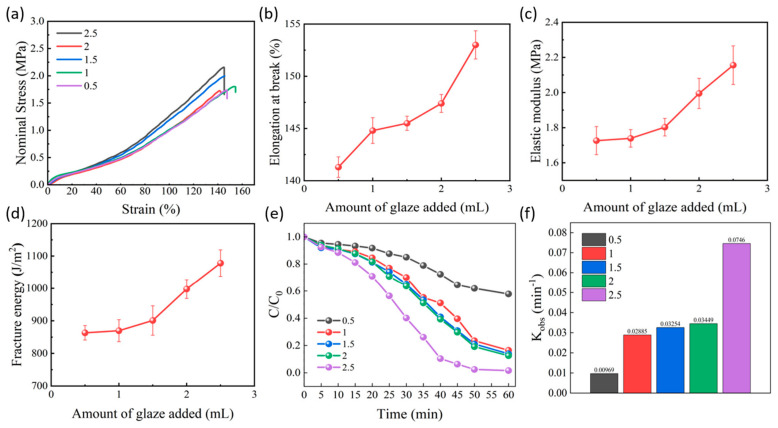
Stress strain curves (**a**), elongation at break (**b**), elastic modulus (**c**), fracture energy (**d**), degradation curve of orange G over time (**e**), and fitted reaction rate constant (**f**) for different glaze addition amounts.

Figure 4a presents the stress–strain curves of the Glaze-CaAlg membranes with different glaze loadings. With an appropriate amount of glaze incorporated, both the tensile strength and strain at break of the membrane increase significantly, indicating that the glaze particles act as physical crosslinking points that enhance the mechanical integrity of the hydrogel network [28]. Figure 4b shows that the elongation at break of the Glaze-CaAlg membrane increases with increasing glaze content. The uniform dispersion of glaze particles in the membrane effectively transfers stress and retards crack propagation, thereby improving ductility. The elastic modulus of the membrane continuously rises with increasing glaze content, with a notable enhancement observed at a loading of 2.5 mL (Here, a loading of 2.5 mL indicates a glaze-to-water ratio of 1:7, with the volume subsequently adjusted to prepare a 2.5 wt.% sodium alginate solution; a detailed explanation is provided in the membrane preparation procedure section at the end of this paper). The rigid glaze particles restrict the movement of polymer chains in the hydrogel membrane, increasing its stiffness. Fracture energy reflects the energy required to resist fracture. Figure 4d demonstrates that the fracture energy reaches its maximum at a glaze loading of 2.5 mL, at which the membrane exhibits both high strength and high toughness.

Figure 4e shows the degradation curves of 10 ppm Orange G solution by Glaze-CaAlg membranes with different glaze loadings. Membranes with varying glaze contents exhibited distinct degradation rates. With loadings of 0.5–2 mL, the degradation efficiency remained below 75% within 45 min due to insufficient active sites [29]. When the loading was increased to 2.5 mL, the degradation efficiency reached 93.7% within 45 min. Figure 4f presents the reaction rate constants obtained by fitting the degradation curves to a pseudo-first-order kinetic model. It can be seen that the reaction rate constant k is positively correlated with the glaze loading, reaching an optimal value at a loading of 2.5 mL. This trend is consistent with the variation in mechanical properties, indicating that an appropriate loading not only ensures catalytic activity but also maintains the structural stability of the membrane. Based on the mechanical and catalytic performance data, all subsequent experiments were carried out with a glaze loading of 2.5 mL. A concentration of 10 ppm Orange G is representative of the actual dye concentration in printing and dyeing wastewater; therefore, this concentration was used for all tests except when the effect of pollutant concentration itself was specifically investigated.

### 2.4. Effect of PMS Concentration on OG Removal Efficiency

**Figure 5:**
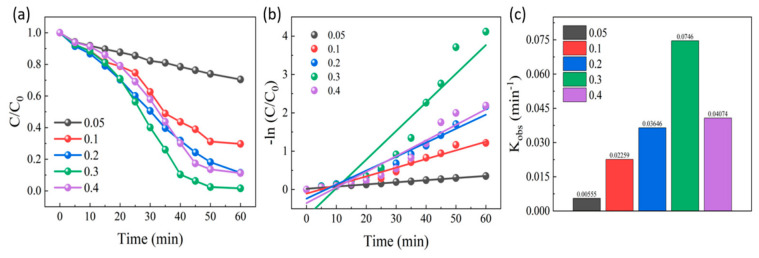
OG removal efficiency by Glaze-CaAlg membrane under different PMS concentrations (**a**), pseudo-first-order kinetic fitting curves (**b**), and reaction rate constants (**c**).

Figure 5a–c illustrate the effect of different PMS concentrations on the degradation of Orange G. As the PMS concentration increased from 0.05 mmol/L to 0.4 mmol/L, a significant difference in the removal efficiency of Orange G was observed. Within the same reaction time, a PMS concentration of 0.3 mmol/L exhibited the optimal degradation performance. The increase in PMS concentration suggests that more persulfate is activated to generate sulfate radicals (SO_4_^−^·) and hydroxyl radicals (·OH), thereby accelerating the oxidative decomposition of dye molecules [30]. When the PMS concentration exceeded 0.3 mmol/L, radical scavenging reactions were induced by the excess PMS [31]. This trend is clearly reflected in the reaction rate constants. Specifically, the k value gradually increased with rising PMS concentration, reaching a minimum at 0.05 mmol/L and a maximum at 0.3 mmol/L, but decreased when the concentration exceeded 0.3 mmol/L. Therefore, considering both cost-effectiveness and efficiency, the suitable PMS concentration in this system is 0.3 mmol/L.

### 2.5. Effect of OG Initial Concentration on Its Removal Efficiency

**Figure 6:**
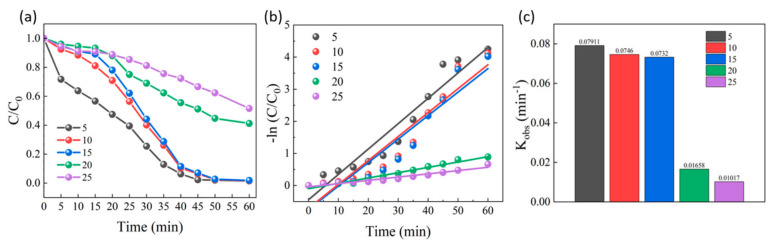
Effect of initial OG concentration on the removal efficiency of Glaze-CaAlg membrane (**a**), pseudo-first-order kinetic fitting curves (**b**), and reaction rate constants (**c**).

Figure 6a presents the effect of different initial Orange G (OG) concentrations on the degradation efficiency. As the initial OG concentration increased from 5 ppm to 25 ppm, the removal efficiency decreased within the same reaction time. At an initial concentration of 5 ppm, nearly 100% removal was achieved after 40 min of reaction. At concentrations of 10 ppm and 15 ppm, over 90% removal was achieved within 45 min. When the concentration was further increased, the catalytic efficiency dropped significantly. This is likely because the number of active metal sites in the glaze is limited, and the stable molecular structure of OG requires longer reaction time for effective degradation. Figure 6b shows the linear fitting of the degradation data under different initial concentrations using a pseudo-first-order kinetic model. The linear relationship between the vertical axis and time confirms that the catalytic oxidation process follows pseudo-first-order reaction kinetics. As the initial OG concentration increased, the k value generally decreased. The k value was 0.07911 min^−1^ at 5 ppm and dropped to 0.01017 min^−1^ at 25 ppm. Under fixed PMS concentration and glaze loading, a higher pollutant concentration requires more catalytic active sites and sufficient radical generation to achieve complete degradation [32]. Therefore, when facing more complex water conditions, increasing the glaze loading in the Glaze-CaAlg membrane could be a solution.

### 2.6. Effect of MIT Initial Concentration on Its Removal Efficiency

**Figure 7:**
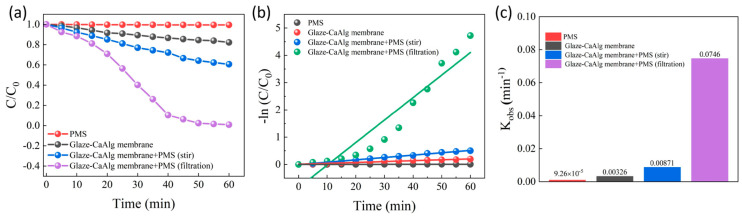
Degradation efficiency of OG in different systems (**a**), pseudo-first-order kinetic fitting curves (**b**), and reaction rate constants (**c**).

Figure 7a shows the degradation of OG in different systems. The system containing PMS alone exhibited no degradation of OG. In the system containing only the Glaze-CaAlg membrane, the degradation efficiency of OG was only 14.6% within 45 min, which is attributable to limited adsorption by the composite membrane. When both the Glaze-CaAlg membrane and PMS were added under manual stirring, the degradation efficiency reached 33.4% within the same time period. In contrast, under pressure-driven cross-flow filtration mode, the degradation efficiency was as high as 93.7%. This experiment validates the superiority of the cross-flow filtration mode. Within the pressure field, the rapidly circulating water contacts the membrane surface, activating PMS to generate a large number of reactive species, thereby degrading OG in a shorter time. As shown in Figure 7c regarding the kinetic reaction rate constants, the rate constant under cross-flow filtration mode was significantly higher than that under manual stirring, reaching 8.56 times that of the manually stirred system.

### 2.7. Active Species Analysis

**Figure 8:**
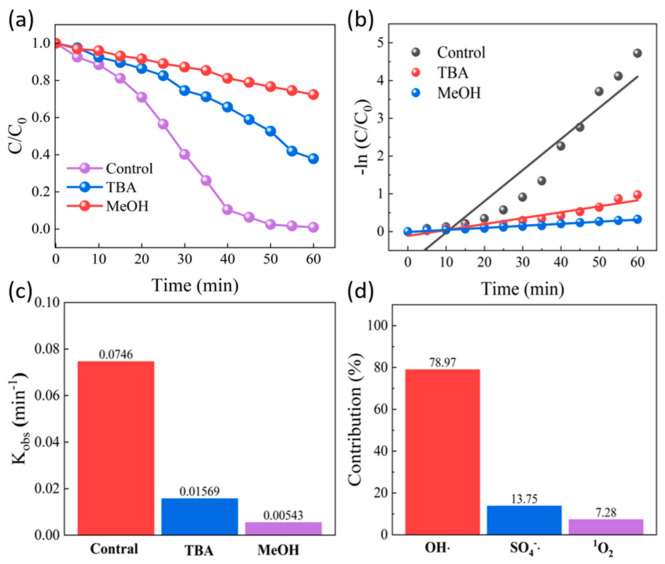
Quenching experiment of active species (**a**) and dynamics fitting curve of quenching experiment (**b**). (**c**) calculates the rate constants of each reaction after fitting. The contribution of each reactive species was calculated, as shown in (**d**).

As shown in Figure 8a, inthe control group without any quencher, our previous experiments have demonstrated that the Glaze-CaAlg membrane can effectively activate PMS to degrade OG. Upon the addition of tert-butanol (TBA), a quencher for hydroxyl radicals (·OH), the degradation curve flattened, and the degradation efficiency decreased to 41.10% within 45 min, indicating that the degradation reaction was hindered after ·OH was significantly suppressed. When methanol (MeOH), which scavenges both hydroxyl radicals (·OH) and sulfate radicals (SO_4_^−^·), was added, the degradation efficiency further decreased to 21.10% within 45 min. These results confirm the dominant role of the radical pathway. Figure 8b shows the fitting curve of the quenching experiment.

Figure 8c calculates the rate constants of each reaction after fitting. After fitting, the rate constant k of the control group was 0.0746 min^−1^. Upon the addition of TBA, the k value decreased to 0.01569 min^−1^, a reduction of approximately 79%. After adding MeOH, the k value further decreased to 0.00543 min^−1^, a reduction of 93%. Based on these results, the contribution of each reactive species was calculated, as shown in Figure 8d, the contribution of ·OH was the highest at 78.97%, followed by SO_4_^−^· at 13.75%, and singlet oxygen (^1^O_2_) at only 7.28%. According to these calculations, ·OH is the dominant reactive species, SO_4_^−^· is secondary, and the non-radical pathway contributes minimally. This indicates that in the Glaze-CaAlg membrane/PMS activation system, the multimetallic sites (Co, Mn, Fe, etc.) mainly react with PMS to generate ·OH and SO_4_^−^· radicals, achieving efficient degradation of Orange G, with ·OH playing the primary role.

### 2.8. Reusability of Glaze-CaAlg Membrane

**Figure 9:**
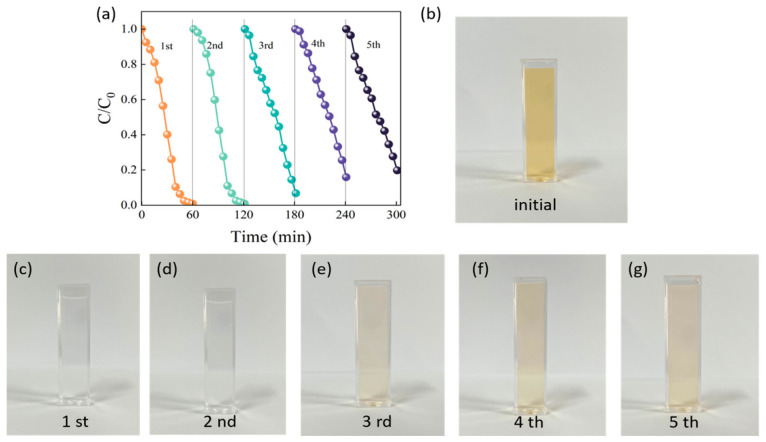
Effect diagram of cyclic degradation of OG by Glaze CaAlg membrane for five times (**a**), initial 10 ppm OG digital photo (**b**), and OG digital photos after the first to fifth degradation (**c**–**g**).

Figure 9a presents the performance of the Glaze-CaAlg membrane over five consecutive degradation cycles, with a reaction time of 60 min per cycle. After each cycle, only the Orange G (OG) solution was replaced, while the membrane was kept unchanged. During the first two cycles, the removal efficiency approached 100% at 60 min. In the third cycle, the degradation efficiency decreased slightly, yet still reached 93.2% at 60 min. The degradation efficiencies in the fourth and fifth cycles were 84.1% and 80.2%, respectively. Figure 9b–g show digital photographs of the OG solution before and after degradation in the cycling experiments. The initial 10 ppm OG solution exhibited a distinct orange-yellow color; after the first two cycles, the solution became nearly colorless and transparent, indicating efficient OG degradation. After the third cycle, the solution appeared very pale yellow, while after the last two cycles, a noticeable light orange color remained, which is consistent with the removal efficiencies presented in the degradation curves.

Overall, the Glaze-CaAlg membrane retains a certain degradation capacity after five repeated uses. The primary reason for the decrease in catalytic activity arises from the valence state evolution of Co during the reaction, specifically the transformation from the highly active Co^2+^ to the less active Co^3+^ [33]. Meanwhile, cobalt undergoes irreversible leaching in the oxidative environment, resulting in the loss of active components [34,35]. Additionally, no regeneration treatment was applied to the membrane after each cycle, allowing accumulated adsorbed pollutants to remain on the membrane. Nevertheless, the membrane still exhibits good reusability and holds certain application potential in the treatment of actual printing and dyeing wastewater.

### 2.9. Experiment on the Recycling and Reuse of Glaze

**Figure 10:**
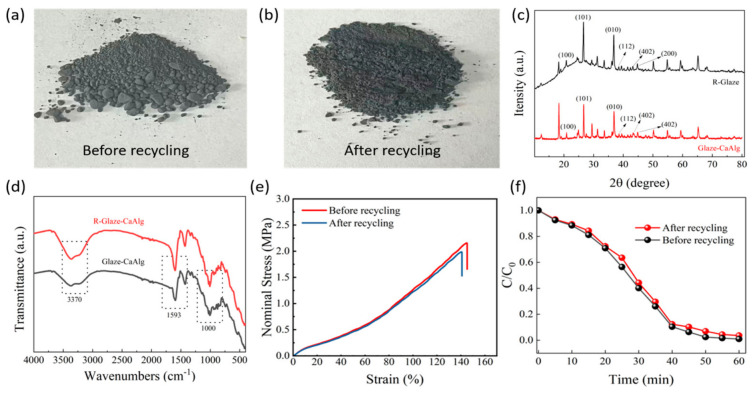
Digital photo of glaze before (**a**) and after recycling (**b**), XRD testing of recycled glaze powder and Glaze-CaAlg membrane (**c**), infrared curve testing of Glaze-CaAlg membrane before and after recycling (**d**), stress strain curves of R-Glaze-CaAlg and Glaze-CaAlg membrane (**e**), and catalytic performance curve of R-Glaze-CaAlg and Glaze-CaAlg membrane (**f**).

Figure 10a,b show a comparison of the morphology of the glaze powder before and after recycling. The overall appearance is similar, and the color of the recycled glaze is slightly darker. Figure 10c shows XRD analysis of the recovered glaze powder and Glaze-CaAlg membrane. The intense peaks at 2θ = 18.35°, 26.65°, 39.54°, and 59.47° are assigned to the ceramic matrix phases. Corresponding to components such as silicon dioxide (PDF card No. 46 1045), kaolin (PDF card No. 16-0606), potassium feldspar (PDF card No. 31-966), etc. The diffraction peaks of 2θ ≈ 36.5° and 42.4° correspond to the (110) and (200) crystal planes, which are characteristic reflections of the cubic CoO phase (PDF card numbers 48-1719). In addition, due to the low content of elements such as Fe and Mn, the diffraction peaks exhibit weaker characteristics. The weak diffraction peaks located at 20.86°, 38.36°, and 43.15° are identified as characteristics of the presence of MnO, Fe_2_O_3_, and Al_2_O_3_. The clear distinction between the ceramic matrix peaks and the metal oxide peaks confirms that the catalytic active phases are well preserved and identifiable within the composite membrane. At the same time, it was also observed that the XRD pattern of the recycled glaze was basically the same as that of the initial membrane. The metal characteristic peaks and ceramic matrix phases are well preserved, with no significant peak shift or formation of new phases. These results confirm that sodium citrate treatment is mild and does not cause any phase transition or structural degradation of active metal oxides in the glaze.

To further verify that the re-fabrication process does not alter the chemical structure of the composite membrane, FTIR spectra of the pristine Glaze-CaAlg membrane and the membrane re-fabricated from recovered glaze (R-Glaze-CaAlg) were compared (Figure 10d). Both spectra exhibit the characteristic absorption bands of calcium alginate. The absence of any significant peak shift or intensity change confirms that the calcium alginate crosslinking structure is successfully reconstructed after the recovery process. The absorption peak of the metal oxygen bond located in the low wavenumber range confirms the existence of the glaze. This FTIR results demonstrate that the sodium citrate recovery procedure does not compromise the chemical integrity of either the hydrogel matrix or the catalytic glaze.

Figure 10e shows the stress–strain curves of the Glaze-CaAlg membrane and the re-fabricated membrane from recovered glaze (R-Glaze-CaAlg). The stress at maximum strain for the original membrane is approximately 0.19 MPa, while that of the recycled membrane is about 0.18 MPa, a decrease of only about 5%. When re-fabricating the membrane, the recovered glaze can still be uniformly dispersed in the calcium alginate hydrogel network, retaining its original particle size and maintaining the mechanical integrity of the composite membrane. Figure 10f presents a comparison of the degradation performance of Orange G between the Glaze-CaAlg membrane and the membrane re-fabricated from recovered glaze. The two degradation curves are very close, indicating that the recovery process did not impair the catalytic activity of the glaze. This is attributed to the mild recovery procedure using sodium citrate for the transition metal oxides (Co, Mn, Fe, etc.) in the glaze, which effectively avoided oxidation or deactivation of the metal sites.

The sodium citrate recovery method enables efficient and mild separation and recovery of raw materials in the calcium alginate membrane-supported catalyst system. In this study, the recovered glaze, when re-fabricated into membranes, retained almost the full catalytic degradation capacity and mechanical properties of the original membrane. This provides a feasible strategy for the recycling of catalytic membrane materials in printing and dyeing wastewater treatment, significantly reducing material costs and minimizing solid waste generation.

## 3. Conclusions

This study successfully fabricated a calcium alginate hydrogel catalytic membrane loaded with glaze. The glaze contains multiple components, including cobalt oxide, manganese oxide, iron oxide, potash feldspar, quartz, and kaolin. We systematically evaluated its mechanical properties, performance in activating peroxymonosulfate (PMS) for Orange G degradation, reusability, and the potential for glaze recovery and reuse. The results show that an appropriate glaze loading significantly enhances the tensile strength, elastic modulus, and fracture energy of the composite membrane, exerting a physical crosslinking reinforcement effect. Regarding catalytic degradation, the composite membrane efficiently activates PMS to generate hydroxyl radicals (·OH) and sulfate radicals (SO_4_^−^·). In this study, the contribution of ·OH was approximately 79%, and that of SO_4_^−^· was approximately 14%, together achieving effective removal of Orange G. The degradation rate peaked at a PMS concentration of 0.3 mmol/L. After five cycles of reuse, the composite membrane still maintained a removal efficiency above 80%, demonstrating good reusability. After mild recovery of the glaze using sodium citrate and re-fabrication of the membrane, the resulting composite membrane exhibited catalytic degradation and mechanical properties highly consistent with those of the original membrane, confirming the effectiveness of the recovery strategy. In summary, the Glaze-CaAlg membrane combines good mechanical strength, high PMS activation efficiency, reusability, and glaze recoverability, providing an environmentally friendly and economically viable catalytic membrane material for advanced oxidation treatment of refractory organic dyes in printing and dyeing wastewater.

## 4. Experiment

### 4.1. Materials and Methods

Glaze was purchased from Guanzhao Xuan (Tianjin, China) Cultural Communication Co., Ltd. Sodium alginate (NaAlg, food grade), calcium chloride (CaCl_2_, AR), methanol (MeOH, AR), tert-butanol (TBA, AR), potassium persulphate (PMS, AR), and Orange G (OG, AR) were all purchased from Shanghai Aladdin Reagent Co., Ltd (Shanghai, China).

### 4.2. Preparation of Glaze-CaAlg Membrane

**Figure 11:**
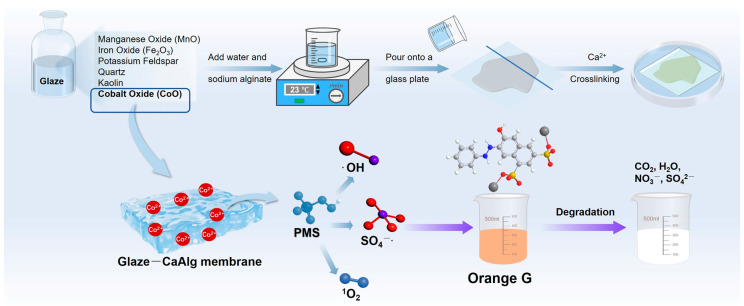
Schematic illustration of the preparation and application of Glaze-CaAlg membrane.

Figure 11 presents a schematic diagram of the preparation process and application of the Glaze-CaAlg membrane.

A certain amount of glaze dispersion was added to a beaker and uniformly mixed with deionized water at a glaze-to-water ratio of 1:7, with continuous stirring until homogeneous dispersion was achieved. Then, a calculated amount of sodium alginate was slowly added to obtain a final concentration of 2.5 wt.% sodium alginate relative to the total solution volume. The mixed solution was continuously stirred using a stirrer, and the beaker was removed after homogeneous mixing was achieved. To prevent the formation of bubbles within the casting solution caused by high-speed stirring, the beaker was allowed to stand for 5 h before further processing. During membrane fabrication, an appropriate volume of the casting solution was poured onto a flat, dry glass plate and spread using a glass rod wrapped with a fixed copper wire. The glass plate was then immersed in a calcium chloride solution for crosslinking to obtain the Glaze-CaAlg membrane.

### 4.3. Characterization of Glaze-CaAlg Membrane

The surface morphology of the Glaze-CaAlg membrane was characterized using a thermal field emission scanning electron microscope (TFE-SEM, Model B110G500, ZEISS, Oberkochen, Germany). The SEM analysis was performed at an accelerating voltage of 10.0 kV, with a working distance of 8.5 mm. Prior to imaging, all samples were sputter-coated with gold to improve surface conductivity. Capture images at different magnifications using secondary electrons. Prior to testing, the membrane samples were cut to appropriate sizes and dried. Subsequently, energy-dispersive X-ray spectroscopy (EDS, Ultim Max 65, Oxford, UK) was conducted on the membrane surface via multi-point scanning to acquire elemental distribution maps and semi-quantitative compositional data. Phase identification was performed using X-ray diffraction (XRD, D8 DISCOVER, Bruker, Germany) under the following operating conditions: accelerating voltage of 60 kV, emission current of 80 mA, step-scanning range of 2θ = 5–90°, and scanning rate of 5°/min. All diffraction patterns were subjected to background subtraction.

### 4.4. Mechanical Properties Testing

The mechanical properties of the Glaze-CaAlg membrane were measured using a fiber tensile tester (LLY06F, Laizhou Electronic Instrument Co., Ltd., Laizhou, China). Prior to testing, the membrane was cut into rectangular strips, and its thickness was measured. For each type of membrane, ten tensile tests were conducted at room temperature with a stretching rate of 10 mm/min, and the results were averaged. The elongation at break (λ) was calculated according to Equation (1) [36]:

(1) $$\;\lambda =(L-L_{0})/L_{0}\times 100\%$$

In the above equation, *L*_0_ is the initial length of the Glaze-CaAlg membrane (mm), and *L* is the length after tensile testing (mm).

### 4.5. Catalytic Degradation Performance Testing

The catalytic degradation of OG was kinetically analyzed using a pseudo-first-order model, with the rate constant k determined by fitting to Equation (3) and the removal efficiency R calculated according to Equation (2). For this purpose, a custom membrane cell with an effective filtration area of 19.6 cm^2^ was employed. The experimental procedure was as follows: the membrane was first pre-pressurized with deionized water at 0.1 MPa for 10 min to clean the surface; subsequently, the feed was switched to OG solution, and PMS was added to initiate degradation. During the reaction, samples were taken at 1-min intervals and their absorbance was measured at 475 nm using a UV–Vis spectrophotometer. All data reported are the averages of three independent replicate experiments [36].

(2) $$R=(C_{0}-C)/C_{0}\times 100\%$$

(3) $$-ln(C/C_{0})=kt$$

where *C*_0_, *C*, *k*, and *t* represent the initial pollutant concentration (mg/L), the residual concentration at time *t* (mg/L), the reaction rate constant (min^−1^), and the elapsed reaction time (min), respectively.

### 4.6. Quenching Experiment of Active Species

Based on the corresponding kinetic equations, the contribution of each reactive species to the catalytic process can be quantitatively evaluated. To obtain the necessary data, quenching experiments were conducted under identical catalytic conditions: at the beginning of the reaction, 24.30 mL of methanol and 57.38 mL of tert-butanol were sequentially added to the system, followed by the introduction of 0.3 mmol/L PMS to initiate quenching. By comparing the rate variations with and without the quenchers, the respective contributions of active species such as ·OH and SO_4_^−^· were determined.

(4) $$\lambda \left[\cdot \mathrm{OH}\right]=(k_{0}\;-\;k_{1})/k_{0}\;\times \;100\%$$

(5) $$\lambda \left[SO_{4}{-}^{\cdot }\right]=\left(k_{1}\;-\;k_{2}\right)/k_{0}\;\times \;100\%$$

(6) λO21=100– λ·OH− λSO4−·

In these equations, λ[·OH], λ[SO_4_^−^·], and λ[^1^O_2_] correspond to the respective contributions of ·OH, SO_4_^−^·, and ^1^O_2_, while *k*_0_, *k*_1_, and *k*_2_ denote the rate constants measured without quencher, with TBA, and with MeOH, in that order.

### 4.7. Glaze Recycling Experiment

First, approximately 6.5 g of the Glaze-CaAlg membrane was cut into small rectangular pieces using scissors. The pieces were then immersed in 30 mL of 0.2 mol/L sodium citrate solution under continuous stirring for 3 h to completely dissolve the calcium alginate matrix. The solid residue was separated by centrifugation, dried in an oven at 75 °C, and finally ground to obtain the recovered glaze.

## Data Availability

The original contributions presented in this study are included in the article. Further inquiries can be directed to the corresponding author.

## List of Abbreviations

OH	Hydroxyl radicals
CaAlg	Calcium alginate
Glaze-CaAlg	Glaze calcium alginate membrane
EDS	Energy dispersive spectrometer
OG	Orange G
NaAlg	Sodium alginate
TOC	Total organic carbon
PMS	peroxymonosulfate
PS-AOPs	Persulfate-based advanced oxidation processes
ROS	Reactive oxygen species
SO_4_^−^·	Primarily sulfate radicals
SEM	Scanning electron microscope
XRD	X-Ray Diffraction
